# Supplementary material for: YAP/TEAD1 Complex Is a Default Repressor of Cardiac Toll-Like Receptor Genes
Source: Int J Mol Sci. 2021 Jun 22;22(13):6649. doi: 10.3390/ijms22136649 (PMC8268263; doi:10.3390/ijms22136649)
Supplement: Supplementary file 1 [file ijms-22-06649-s001.zip › ijms-1260123-supplementary.pdf]

Supporting Information

YAP/TEAD1 complex is a default repressor of cardiac Toll like receptor genes

*Yunan Gao[a, b], *Yan Sun[a], A. Gulhan Ercan-Sencicek[a, c], Justin S. King[d], Brynn N. Akerberg[d], Qing Ma[d], Maria I. Kontaridis[a], William T. Pu[d], and Zhiqiang Lin[a, #]

a. Masonic medical research institute, 2150 Bleecker St, Utica, NY 13501

b. Department of Cardiology, the Fourth Affiliated Hospital of Harbin Medical University, Harbin, China.

c. Department of Neurosurgery, Program on Neurogenetics, Yale School of Medicine, Yale University, New Haven, CT, USA

d. Boston Children's Hospital, 300 Longwood Ave, Boston, MA, 02115

* These authors contributed equally

# To whom correspondence should be addressed. E-mail: zlin@mmri.edu

Supplementary Material and methods

Animal surgeries

Before surgery, mice were anesthetized in an induction chamber with 2% isofluorane mixed with 0.5 -1.0 L/min 100% O2. During the surgical procedure, Isofluorane anesthesia was maintained at 1.5-2% isofluorane with 0.5 - 1.0 L/min 100% O2. Correct level of anesthesia was verified by applying pressure on the mouse nail bed (toe-pinch reflex). To induce pressure overload stress, standard transverse aortic constriction (TAC) surgery was applied to 6-8 weeks-old male CFW mice with 25-30 gram body weight [1]. 27 Gauge needle was used in TAC operation. Ischemia/ Reperfusion (IR) surgery [2] was applied to 6-8 weeks-old male C57BL/6J mice. The left anterior descending artery (LAD) was ligated for 50 minutes before reperfusion. To reduce pain after surgery, meloxicam (5-10mg/kg) was subcutaneously infused to the mice every 22-24 hours for 72 hours and 48 hours in TAC- and IR-stressed animals, respectively. In TAC or IR surgeries, animals undergoing open-chest surgery but without TAC or LAD ligation were used as corresponding sham controls, respectively. At the end of the study, mice were euthanized with overdose CO_2.

AAV9 packaging and administration

AAV9.cTnT.GFP and AAV9.cTnT.iCre [3] was packaged in 293T cells with AAV9:Rep-Cap and pHelper (pAd deltaF6, Penn Vector Core) and purified and concentrated by gradient centrifugation. AAV titer was determined by quantitative PCR, in which a primer pair amplifying a fragment of the chicken cardiac TnT (cTnT) promoter was used. The primers sequences were : 5'-GCTTTCACATGACAGCATCTGGGG-3'; 5'-CCCAAGCTATTGTGTGGCCT-3'.

For retro-orbital AAV injection, mice were anesthetized with 3% isoflurane. A 30 gauge needle was inserted at a 45° angle to the eye, lateral to the medial canthus, through the conjunctival membrane. The needle was positioned behind the globe of the eye in the retrobulbar sinus. Less than 100 μl of viral solution was injected.

Echocardiography (Echo) measurements

Before being tested, the mouse chest was depilated and the mice were trained for echo for three consecutive days. Briefly, the mice were held in a position to expose the chest, and an artificial plastic probe was put onto the chest to mimic the action of the ultrasound probe. On the third day, conscious mice were subjected to Echo measurements. The ultrasound probe and gel was applied to the chest to obtain echocardiography measurements of the heart. This typically took 5-10 minutes, during which mice were held for approximately one-minute intervals. After completion of the study, the gel was wiped from the chest with a paper towel, and the mouse was returned to normal housing.

TLR4 TSS adjacent region cloning and Luciferase activity measurements

A 613 bp fragment of human TLR4 genomic DNA was amplified with the following primers that contain NheI (primer 1) or BamHI (primer 2) restriction sites. Primer 1, ATAGCTAGCCACT-

TGCTGACACCTTGAGTTCAGAC; primer 2, GCTGGATCCATGG-CTGGGATCAGAGTCCCAG-CCAGGC. The PCR product was then cloned into pGL2 basic vector and named TLR4_Luci. TEAD1 binding motif mutation was introduced with primer 3 that contains a PstI restriction site : GGTCTGCAGGCGTTTTCTTCTTCTAACTTCCTCTCCTGTGACAAAAGAGATAA-CTATTAGAGAAACAAAAGTCCAtccaaCTAAGGT. PCR product of primer 2 and 3 was used to replace the corresponding part of TLR4 genomic sequence in TLR4_Luci reporter, yielding TLR4_Luci_Mut

HEK 293T cells were cultured in 24 well plates for luciferase assay. 100 ng/well indicated plasmids and 10 ng pRLTK internal control vector (Promega) were co-transfected with 1.25 µl Lipofectamine 3000 (Invitrogen). Luciferase activity was measured 24 hours after transfection using the Dual-Luciferase reporter assay system (Promega).

Immunohistochemistry staining

Hearts were fixed in 2% PFA, dehydrated with ethanol, and embedded in paraffin. For chromogenic immunohistochemistry staining on paraffin sections, 8-µm sections were deparaffinized, rehydrated, and antigen exposed by boiling in Target Retrieval Solution (Dako). Primary antibodies used for this study were summarized in supplementary table 2.
Signals were detected using the Anti-Rabbit IgG (alkaline phosphatase) Polymer Detection Kit (Vector Laboratories). Imaging was performed on Keyence microscope.

Supplementary Table 1. qRT-PCR primers

Species	Gene name	Forward	Reverse
Mouse	*Tlr2*	GAGGTGCGGACTGTTTCCTT	AGATTTGACGCTTTGTCTGAGG
Mouse	*Tlr3*	GGTTGACGCACCTGTTCTCT	TGGGTGCAATCCCTGTATCA
Mouse	*Tlr4*	CCTGACACCAGGAAGCTTGA	TCCAGCCACTGAAGTTCTGA
Mouse	*Tlr7*	AATGCACTCTTCGCAGCAAC	CCGTGTCCACATCGAAAACA
Mouse	*Tlr8*	TTGCTGATGCTAAAGCGACC	GAATCCATGACTGAGGGGGC
Mouse	*Yap*	GACCCTCGTTTTGCCATGAA	ATTGTTCTCAATTCCTGAGAC
Mouse	*Tlr4* ChIP PCR primer set 1	CTTGGGAGTCTAACTGAAGCAGTAAG	GACTGAAGACTAGAACAGGAAGAAGG
Mouse	*Tlr4* ChIP PCR primer set 2	TCCAATGGGCCTCTCTTTCC	ACCCAAGGAGCTAAAGGGAT
Mouse	*Il-12a*	GATGACATGGTGAAGACGGC	AGGCACAGGGTCATCATCAA
Mouse	*Il-12b*	GAAGGAGACAGAGGAGGGGT	TTGGGGGACTCTTCCATCCT
Mouse	*Tlr1*	GGTTGTCTTGACGGAACACG	TTCTTCAGAGCATTGCCACA
Mouse	*Tlr5*	TCACTGCATACCTGGTTCCC	TTGACATGCCATGATCCTGCT
Mouse	*Tlr6*	GGTACCGTCAGTGCTGGAAA	TATTAAGGCCAGGGCGCAAA
Mouse	*Tlr9*	ACAGCCCTGACTAGGGACAA	GCAACTCGGGAACCAGACAT
Mouse	*Il1b*	TGTGCAAGTGTCTGAAGCAGCTA	TCAAAGGTTTGGAAGCAGCCCT
Mouse	*Rela*	ATAAATGCGAGGGGCGCTCA	GGATCCCCAGGTTCTGGAAG
Mouse	*Gapdh*	CAGGTTGTCTCCTGCGACTT	GGCCTCTCTTGCTCAGTGTC
Mouse	*Ccl2*	GTTGGCTCAGCCAGATGCA	AGCCTACTCATTGGGATCATCTTG
Mouse	*Cyr61*	GCTCAGTCAGAAGGCAGACC	GTTCTTGGGGACACAGAGGA

Supplementary Table 2. Primary antibody list

Antigen	Host	Vendor (Cat #)	Usage (dilution)
β-actin	Mouse	Santa Cruz (sc-47778)	Western blot (1:1000)
RelA	Rabbit	Santa Cruz (sc-372)	Western blot (1:1000)
TLR4	Mouse	Santa Cruz (sc-13591)	Western blot (1:1000)
TLR4	Rabbit	Bioss (bs-1021R)	IHC (1:200)
Mac-3	Rat	BD (553322)	IHC (1:100)
IL-12β	Rabbit	Bioss (bs-10641R)	IHC (1:200)
IL-1β	Rabbit	CST (D4T20)	IHC (1:200)
GAPDH	Mouse	Santa Cruz (sc-47724)	Western blot (1:1000)
Ly6G	Rat	BD (551459)	IF (1:200)
YAP	Rabbit	Proteintech (13584-1-AP)	Western blot (1:1000)

References

[1] Rockman, H.A., Ross, R.S., Harris, A.N., Knowlton, K.U., Steinhelper, M.E., Field, L.J., Ross, J. and Chien, K.R. (1991) Segregation of atrial-specific and inducible expression of an atrial natriuretic factor transgene in an in vivo murine model of cardiac hypertrophy. *Proc Natl Acad Sci U S A* **88**, 8277-8281.

[2] Chen, J., Ma, Q., King, J.S., Sun, Y., Xu, B., Zhang, X., Zohrabian, S., Guo, H., Cai, W., Li, G., Bruno, I., Cooke, J.P., Wang, C., Kontaridis, M., Wang, D.Z., Luo, H., Pu, W.T. and Lin, Z. (2020) aYAP modRNA reduces cardiac inflammation and hypertrophy in a murine ischemia-reperfusion model. *Life Sci Alliance* **3**,

[3] Lin, Z., Zhou, P., von Gise, A., Gu, F., Ma, Q., Chen, J., Guo, H., van Gorp, P.R., Wang, D.Z. and Pu, W.T. (2015) Pi3kcb links Hippo-YAP and PI3K-AKT signaling pathways to promote cardiomyocyte proliferation and survival. *Circ Res* **116**, 35-45.

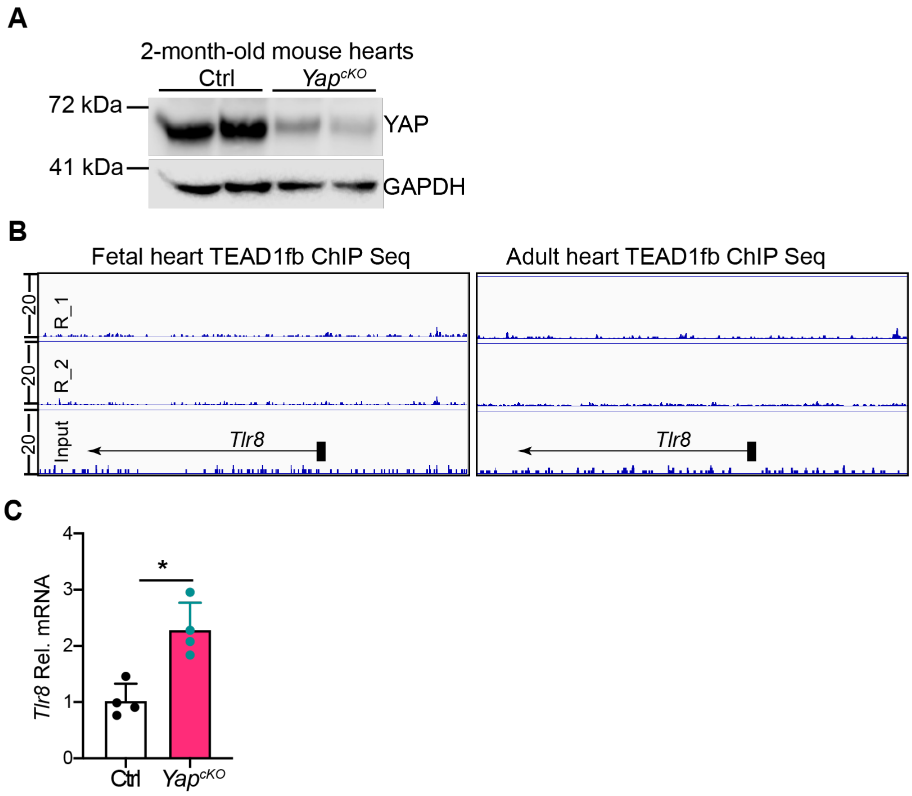

Supplementary Figure 1. YAP/TEAD1 complex regulation of cardiac TLR genes

A. YAP western blot. Total protein extracted from 2-month-old mouse hearts were used for western blot. **B.** Genome browser view showing no obvious TEAD1 ChIP Seq peak around *Tlr8* locus in both fetal and adult hearts. **C**. qRT-PCR measurement of *Tlr8* mRNA level. *Tlr8 mRNA* level was normalized to *Gapdh*. Student t test, *, P<0.05. N=4.

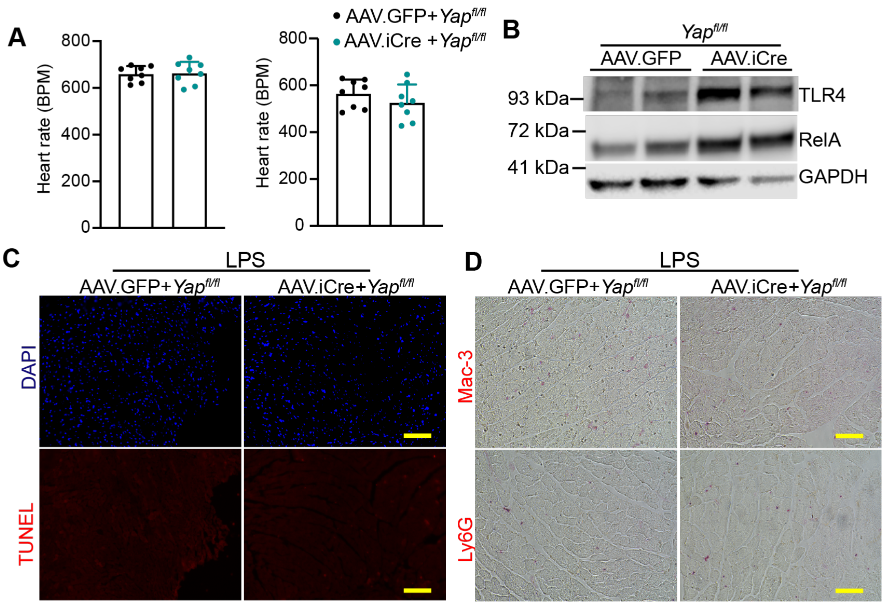

Supplementary Figure 2. Short period of LPS treatment does not induce CM apoptosis.

A. Heart rate before and after LPS treatment. **B.** TLR4 and RelA western blot.

C. TUNEL staining of LPS treated myocardium. **D**. Immunohistochemistry staining of LPS treated myocardium. Mac-3 and Ly6G were used to visualize macrophages and neutrophils, respectively. B, C, hearts were collected for analysis 6 hours after LPS treatment. Scale bar = 50 μm.
